# A new “sweet” ligand for Toll-like receptor 4

**DOI:** 10.18632/oncotarget.4562

**Published:** 2015-06-22

**Authors:** Tomas Deierborg, Miguel A. Burguillos

**Affiliations:** Queen Mary University of London, London, United Kingdom

**Keywords:** immunology and microbiology section, immune response, immunity

One of the common features found in many neurodegenerative disorders such as Parkinson's and Alzheimer's diseases or during stroke, is an ongoing neuroinflammatory response driven by glia (microglia and astrocytes) and immune cells from the peripheral immune system (monocytes, neutrophils, T cells, … ). While a “healthy” activation of the neuroinflammatory response is beneficial through protecting us against viral or bacterial infection or by facilitating the removal of cell debris, an uncontrolled response is however related with neurotoxicity and poorer neurological outcome. Although the neuroinflammatory response depends on the “crosstalk” between these different cell types, microglia cells has been shown to play prominent roles during the pathogenesis of PD, AD and MS. (references found in [1]).

The initiation of the inflammatory response commences at the plasma membrane, through the binding of different ligands to pattern recognition receptors (PRRs). Among all the different PPRs, Toll-like receptors (TLRs) have been shown to play a role in the initiation of the innate immune response against several inflammatory stimuli and not only in the peripheral immune system but also in the CNS (especially in the case of TLR-4) [2]. In 1996 Lemaitre and colleagues [3] discovered in Drosophila that Toll and other genes from the dorsal group played a role in innate immune responses to pathogenic fungi and bacteria. Since then, considerable efforts have been placed aiming uncovering the mechanisms triggered upon TLR activation. One of the most widely used ligand to study the intracellular mechanisms triggered by the dimerization of TLR-4 is the lipopolysaccharide of Gram negative bacteria (LPS). Despite the great advances that the use of LPS has brought in the understanding of the mechanisms underlying TLR-4 activation, its relevance during process of “sterile inflammation” [4], where TLR4 has been shown to play an important role, is questionable. Lately many groups are trying to identify new endogenous TLR-4 ligands that can be relevant in conditions of sterile inflammation such as in stroke. Since then several molecules or proteins have been identified as endogenous TLR ligands.

In our study [5] we have discovered a new and endogenous TLR-4 ligand, galectin-3, that is highly expressed and released by microglial cells during the neuroinflammatory response in two different neuroinflammatory models including brain ischemia and intranigral injection of LPS. We observed that galectin-3 associates with TLR-4 though its carbohydrate recognition domain (CRD). We demonstrate that galectin-3 is capable to induce a neuroinflammatory response in microglial cells that can be abrogated when the levels of TLR-4 are decreased. We also found evidence of interaction of galectin-3 with TLR-4 in some patients who had suffered ischemic stroke.

The galectin family, of which galectin-3 is a member, is involved in several processes including activation of apoptosis, induction of the differentiation process, control of the nuclear splicing of pre-mRNA and the regulation of the inflammatory response. Among the 15 members of the galectin family, galectin-3 is the only known member of the chimera-type, comprising a C-terminal CRD and N-terminal non-CRD for carbohydrate binding. The fine specificity of its carbohydrate recognition domain (CRD), enables galectin-3 to have different affinity for targets that other galectins either cannot bind or do but with a much lower apparent affinity. Besides its specificity towards different targets, galectin-3 function also depends on its location in the cell (galectin-3 has been shown to be in the nucleus, cytosol, in the membrane or secreted).

How does galectin-3 activate TLR-4? Galectin-3 has a great affinity for β-galactoside and TLR-4 contains in its structure β-galactosides where galectin-3 could bind. Galectin-3 could promote the dimerization of TLR-4 due to its ability to form oligomers with other galectin-3 that are already bound to another TLR-4. Another possibility could be that galectin-3 might induce TLR-4 internalization, a mechanisms necessary for full TLR4 activation [6]. Recently it has been shown that galectin-3 is involved in the internalization of several receptors, including CD44 by binding to them and promoting their clathrin-independent endocytosis [7].

Although in our paper we focused on the galectin-3 protein released from microglia cells, galectin-3 expression can be found as well in other cell types such as for instance in monocytes. Consequently the implications of galectin-3 acting as a ligand for TLR-4 can be important not only in neurodegenerative disorders but also in other diseases outside the CNS where there is a strong inflammatory component as for instance inflammatory bowel disease where monocytes and galectin-3 are known to play a role in the pathology.
